# Effect of different types of anesthesia on intraoperative blood glucose of diabetic patients: A PRISMA-compliant systematic review and meta-analysis

**DOI:** 10.1097/MD.0000000000006451

**Published:** 2017-03-31

**Authors:** Xueqiong Li, Jinjing Wang, Kang Chen, Yijun Li, Haibin Wang, Yiming Mu, Yaolong Chen

**Affiliations:** a Department of Endocrinology, Chinese PLA General Hospital, Beijing; b Department of Gerontology, First Affiliated Hospital of Kunming Medical University, Kunming; c Affiliated Hospital of Academy of Military Medical Sciences (PLA 307 Hospital); d Department of Endocrinology, Chinese PLA General Hospital, Beijing; e Evidence-Based Medicine Center, Basic Medical Sciences, Lanzhou University, Lanzhou, China.

**Keywords:** anesthesia, blood glucose control, diabetes, meta-analysis

## Abstract

**Background:**

Systematic review which analyzes the impact of different anesthesia on intraoperative blood glucose levels of diabetes patients.

**Methods:**

We searched Medline (via PubMed), Embase, Cochrane Library, Web of Science, Wangfang, CNKI, and CBM database through June 2016, included in randomized controlled trial (RCT), about different anesthesia on intraoperative blood glucose levels in patients with diabetes. Two researchers in 1 group independently screened literatures with eligibility criteria, extracted information, and used RevMan5.3 software to perform meta-analysis.

**Results:**

We included 11 trials and performed the meta-analysis with 10 trials. The meta-analysis results suggested that compared with general anesthesia, the combined general-epidural anesthesia has a better glycemic control in intraoperative blood glucose levels (WMD −1.26, 95% confidence interval [CI] −1.77 to 0.76), the epidural anesthesia had no significant effects compared with general anesthesia (WMD −0.74, 95% CI 4.41–2.92), and the combined spinal-epidural anesthesia had no significant effects compared with epidural anesthesia (WMD −0.28, 95% CI −1.02 to 0.46). One study suggested that compared with epidural anesthesia, the combined general-epidural anesthesia can lower blood glucose levels

**Conclusion:**

Existing evidence showed that compared with general anesthesia, the combined general-epidural anesthesia has a better glycemic control in intraoperative blood glucose levels.

## 1 Introduction

Diabetes mellitus (DM) is a multisystem metabolic disease, and the number of diabetic patients increased sharply in recent years.^[1]^ A study showed 2% to 4% surgical patients had diabetes.^[2]^ Perioperative patients with diabetes could lead to a sharp increase of blood glucose, causing the increased incidence of diabetic acute complications and infections, delayed wound healing, and postoperative mortality.^[3,4]^ Therefore, discussing the better type of anesthesia and taking glycemic control were necessary. This study will review the impact of different types of anesthesia on intraoperative blood glucose levels of diabetic patients systematically, and provide the evidence to support the choice of anesthesia.

## 2 Materials and methods

### 2.1 Search methods

A search of Medline (via PubMed), Embase, Cochrane Library, Web of Science, Wanfang, CNKI, and CBM databases was conducted to identify related studies from inception of each database through June 2016. We also searched WHO International Clinical Trials Registry Platform (ICPTR) as a supplement. Moreover, we did not limit the language. Detailed retrieval strategies were shown in Appendix 1.

### 2.2 Eligibility criteria

The inclusion criteria were as follows: RCT; diabetic patients undergoing operations with anesthesia; intervention group and control groups used general anesthesia, epidural anesthesia, subarachnoid (spinal) anesthesia, or combined anesthesia; reporting the intraoperative blood glucose levels as the outcomes; language of publications was Chinese or English.

### 2.3 Selection of studies and assessment of risk of bias

Two reviewers independently screened titles, abstracts, and the full texts of included studies, and the disagreement was solved by discussion or consultation with a third researcher. Two reviewers (X.L., J.W.) used the Cochrane risk bias assessment tools^[5]^ to assess the quality of included literatures, consisting of 7 aspects: random sequence generation; allocation concealment; blinding of participants and personnel; blinding of outcome assessment; incomplete outcome data; selective reporting; and other bias.

### 2.4 Data extraction

Data extraction was undertaken independently by 2 reviewers (X.L., J.W.) using standard data extraction templates with the following information: basic information (publication year, first author, institution, journal), and blood sugar level of intervention group and control groups. Also, we checked each other's information.

### 2.5 Statistical analysis

For continuous outcomes, we calculated mean differences and 95% confidence intervals (CIs). For dichotomous data, we calculated odd ratios (ORs) and 95% CIs. We identified heterogeneity by using *Q* test (*P* < 0.05, suggesting the existence of heterogeneity). We also specifically examined heterogeneity employing the I^2^ statistics that is being used to quantify the inconsistency across studies, where an I^2^ statistic of 75% and more indicates a considerable level of inconsistency. We summarized data statistically if they were available, sufficiently similar, and of sufficient quality. We performed statistical analyses according to the statistical guidelines referenced in the newest version of the Cochrane Handbook for Systematic Reviews of Interventions.^[5]^ When there was an obvious or significant heterogeneity, the sensitivity analysis would be used to investigate the sources of the heterogeneity. Statistical analysis was performed using RevMan 5.3 software.

### 2.6 Grading of quality of evidence

The Grading of Recommendations Assessment, Development, and Evaluation (GRADE)^[6–11]^ was used to assess the quality of evidence for each outcome. The criteria were mainly considered: risk of bias, indirectness, inconsistency, imprecision, and publication bias. The quality of evidence for each outcome was graded as high, moderate, low, and very low. Finally, we presented the results of quality of evidence for each outcome through summary of finding table.

The study protocols were approved by the Hospital Ethics Committee.

## 3 Results

### 3.1 Results of the search

**Figure 1:**
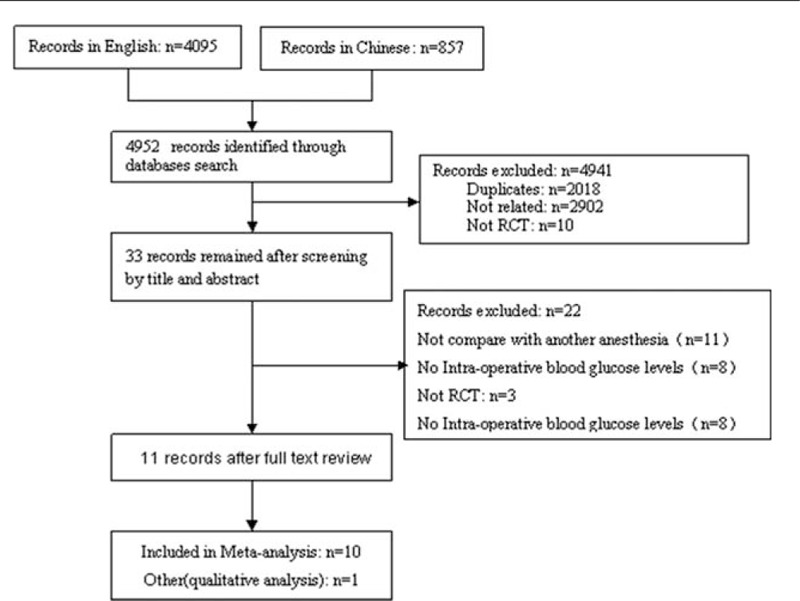
Chart of study selection.

There were a total of 4952 records, 3795 were English, and 857 were Chinese. Also, 4941 were excluded. Finally, the included RCTs were 11.^[12–22]^ The research process was shown in Fig. 1.

### 3.2 Baseline characteristics of included studies

The basic characteristics of the 11 studies are summarized in Table 1.

**Table 1:**
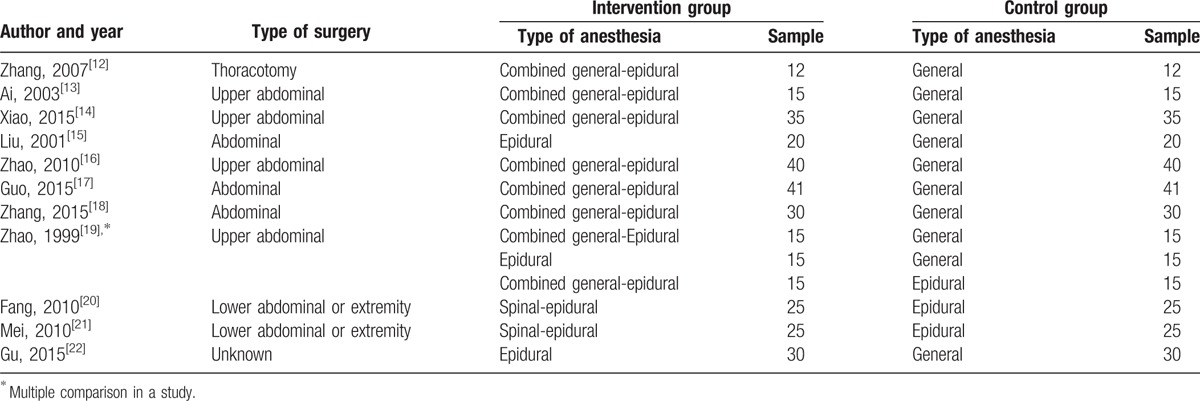
Baseline characteristics of included studies.

### 3.3 Assessment of risk of bias

**Figure 2:**
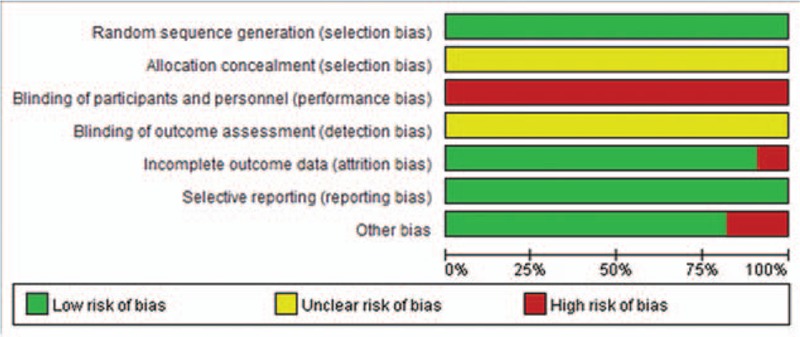
Risk of bias summary.

Among the 11 included studies, risk of bias assessment indicated that all trials reported randomization and no trial blinded patients and researchers. No trial reported allocation concealment and blinding of outcome assessment. Detailed information could be found in Table 2 and Fig. 2.

**Table 2:**
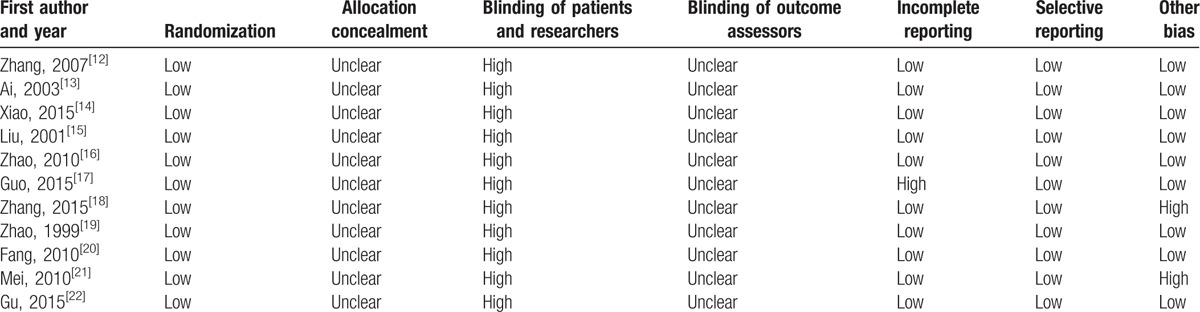
Risk of bias of the included studies.

### 3.4 Results of the meta-analysis

**Figure 3:**
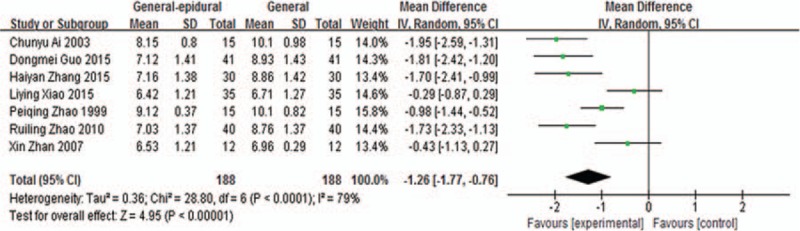
Blood glucose levels comparison by combined general-epidural and general anesthesia.

Meta-analysis of intraoperative blood glucose levels comparison by combined general-epidural and general anesthesia included 7 studies,^[12–14,16–19]^ the results of which are shown in Fig. 3. Using fixed-effects model to perform merger analysis, “combined general-epidural” group had lower blood glucose levels (WMD −1.26, 95% CI −1.77 to 0.76, *P* < 0.00001). The above results suggested that combined general-epidural anesthesia had a better effect on the control of intraoperative blood glucose.

**Figure 4:**
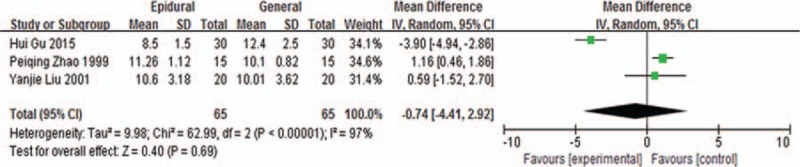
Blood glucose levels comparison by epidural and general anesthesia.

Meta-analysis of intraoperative blood glucose levels comparison by epidural and general anesthesia included 3 studies,^[15,19,22]^ the results of which are shown in Fig. 4. Using random-effects model to perform merger analysis, 2 groups had no statistical differences in blood glucose levels (WMD −0.74, 95% CI −4.41 to 2.92, *P* = 0.69). The above results suggested that epidural anesthesia had no significant effects on the intraoperative blood glucose levels compared with general anesthesia.

**Figure 5:**
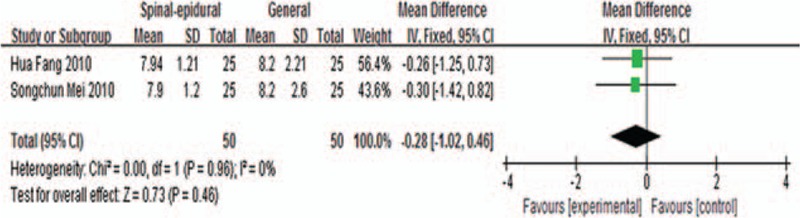
Blood glucose levels comparison by combined spinal-epidural and epidural anesthesia.

Meta-analysis of intraoperative blood glucose levels comparison by combined spinal-epidural and general anesthesia included 2 studies,^[20,21]^ the results of which are shown in Fig. 5. Using fixed-effects model to perform merger analysis, 2 groups had no statistical differences in blood glucose levels (WMD −0.28, 95% CI −1.02 to 0.46, *P* = 0.46). The above results suggested that combined spinal-epidural anesthesia had no significant effects on the intraoperative blood glucose levels compared with general anesthesia.

### 3.5 Blood glucose levels comparison by combined spinal-epidural and general anesthesia

The study by Zhao et al^[19]^ included 30 patients, with 15 patients in combined spinal-epidural anesthesia (intervention) group and 15 in general anesthesia (control) group. The blood glucose levels of experimental group was 9.12 ± 0.37 mmol/L and the levels of control group was 11.26 ± 1.12 mmol/L (*P* < 0.01). The result suggested that combined spinal-epidural anesthesia had a better effect on the control of intraoperative blood glucose compared with general anesthesia.

### 3.6 Publication bias

**Figure 6:**
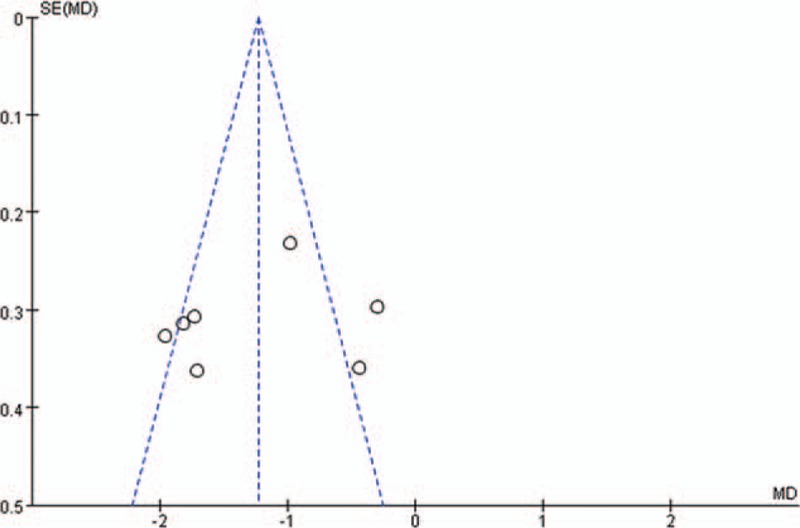
Funnel plot of combined general-epidural comparing with general anesthesia.

In the 7 studies comparing combined general-epidural with general anesthesia on intraoperative blood glucose levels, we made the funnel plot by MD value as X-axis and SE (MD) as Y-axis. The funnel plot was not symmetric and concentrated, and showed that the meta-analyses might have publication bias (Fig. 6).

### 3.7 Assessment of the quality of evidence

Quality of evidence of the above outcomes was presented in Table 3.

**Table 3:**
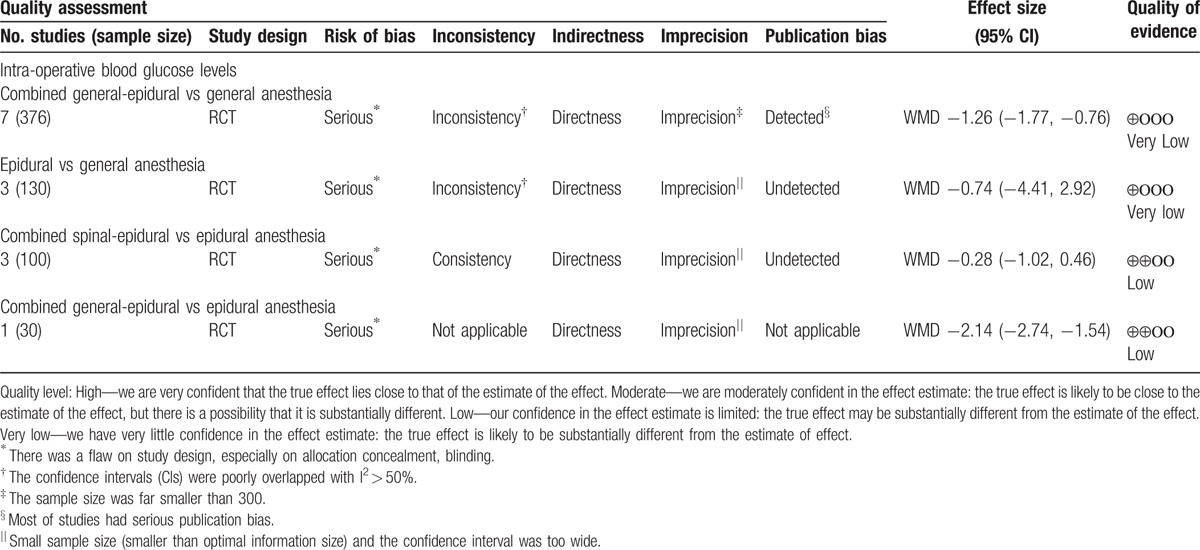
Quality assessment using GRADE approach.

## 4 Discussion

Patients with diabetes would suffer from decreased tolerance of surgical trauma, increased risk, hyperglycaemia and possibility to cause stress. Medium and minor surgery could cause an increase of blood glucose of 1.12 mmol/L on average, as for major surgery it could be 2.05−4.48 mmol/L, and for anaesthetic it could be 0.55−2.75 mmol/L.^[23]^ The rise of perioperative blood glucose levels would increase the risk of infections, which easily led to all kinds of complications and higher surgery risks. So, choice of anesthesia was an important way to assure the stability of blood glucose levels.

Our meta-analyses was performed by included randomized controlled trials about impact of different types of anesthesia on intra-operative blood glucose levels of diabetic patients. The results of meta-analysis showed that compared with single anesthesia, the combined general-epidural anesthesia had a better effect on the control of intraoperative blood glucose levels. But we should pay attention to the advantages and disadvantages of different types of anesthesia, and doctors should choose more appropriate anesthesia according to patients’ conditions and preferences.

The study also had some limitations, which are as follows: small sample in included studies; high risk of bias of the included studies; and big clinical heterogeneity among the included studies. Therefore, there is a need for more high-quality original studies.

## 5 Conclusions

Existing evidence showed that compared with general anesthesia, the combined general-epidural anesthesia has a better glycemic control in intraoperative blood glucose levels.

## Acknowledgments

We acknowledge all clinical researchers of the selected studies and patients related to these studies.
